# Impacts of Interaction of Mental Condition and Quality of Life between Donors and Recipients at Decision-Making of Preemptive and Post-Dialysis Living-Donor Kidney Transplantation

**DOI:** 10.3390/jpm11050414

**Published:** 2021-05-14

**Authors:** Toshiki Hasegawa, Kouhei Nishikawa, Yuko Tamura, Tomoka Oka, Aiko Urawa, Saori Watanabe, Shugo Mizuno, Motohiro Okada

**Affiliations:** 1 Department of Neuropsychiatry, Division of Neuroscience, Graduate School of Medicine, Tsu 514-8507, Japan; t-hasegawa@med.mie-u.ac.jp (T.H.); tomoka-oka@clin.medic.mie-u.ac.jp (T.O.); 2 Organ Transplantation Centre, Mie University Hospital, Tsu 514-8507, Japan; kouheini@clin.medic.mie-u.ac.jp (K.N.); ishoku-rco@clin.medic.mie-u.ac.jp (A.U.); ishoku-rco1@clin.medic.mie-u.ac.jp (S.W.); mizunos@med.mie-u.ac.jp (S.M.); 3 Department of Nephro-Urologic Surgery and Andrology, Mie University Graduate School of Medicine, Tsu 514-8507, Japan; 4 Department of Psychology, Graduate School of Nursing, Mie University, Tsu 514-8507, Japan; y-tamura@nurse.medic.mie-u.ac.jp

**Keywords:** preemptive kidney transplantation, kidney replacement therapy, end-stage renal disease, quality of life, life–work–family balance

## Abstract

Pre-emptive kidney transplantation (PEKT) is considered one of the most effective types of kidney replacement therapies to improve the quality of life (QOL) and physical prognosis of patients with end-stage renal disease (ESRD). In Japan, living-donor kidney transplantation is a common therapeutic option for patients undergoing dialyses (PDKT). Moreover, during shared decision-making in kidney replacement therapy, the medical staff of the multidisciplinary kidney team often provide educational consultation programmes according to the QOL and sociopsychological status of the ESRD patient. In Japan, the majority of kidney donations are provided by living family members. However, neither the psychosocial status of donors associated with the decision-making of kidney donations nor the interactions of the psychosocial status between donors and recipients have been clarified in the literature. In response to this gap, the present study determined the QOL, mood and anxiety status of donors and recipients at kidney transplantation decision-making between PEKT and PDKT. Deterioration of the recipient’s QOL associated with “role physical” shifted the decision-making to PEKT, whereas deterioration of QOL associated with “role emotional” and “social functioning” of the recipients shifted the decision-making to PDKT. Furthermore, increased tension/anxiety and depressive mood contributed to choosing PDKT, but increased confusion was dominantly observed in PEKT recipients. These direct impact factors for decision-making were secondarily regulated by the trait anxiety of the recipients. Unlike the recipients, the donors’ QOL associated with vitality contributed to choosing PDKT, whereas the physical and mental health of the donors shifted the decision-making to PEKT. Interestingly, we also detected the typical features of PEKT donors, who showed higher tolerability against the trait anxiety of reactive tension/anxiety than PDKT donors. These results suggest that choosing between either PEKT or PDKT is likely achieved through the proactive support of family members as candidate donors, rather than the recipients. Furthermore, PDKT is possibly facilitated by an enrichment of the life–work–family balance of the donors. Therefore, multidisciplinary kidney teams should be aware of the familial psychodynamics between patients with ESRD and their family members during the shared decision-making process by continuing the educational consultation programmes for the kidney-replacement-therapy decision-making process.

## 1. Introduction

Chronic kidney disease is a common disease affecting 5∼10% of the population worldwide [1,2,3]. Recently, the prognosis of chronic kidney disease has improved with the development of conservative and replacement therapies. However, the majority of patients with chronic kidney disease remain at risk of progressing to kidney failure (previously end-stage renal disease: ESRD). Indeed, the prevalence of ESRD has increased in recent years [4,5]. There are two types of major therapeutic modalities for ESRD: Dialyses (haemodialysis and peritoneal dialysis) and kidney transplantation [5]. It was established that kidney transplantation in eligible patients with ESRD is a better therapeutic option than long-term dialyses [6,7], due to its association with long-term survival and quality of life (QOL) [8,9,10,11,12]. In particular, several guidelines indicate that every patient with ESRD should be considered for kidney transplantation unless there is an absolute contraindication [6,7,13]. Furthermore, a number of clinical studies have reported that pre-emptive kidney transplantation (PEKT) contributes to a better prognosis among recipients and better survival of the transplant compared to receiving a transplant after dialysis (PDKT) or receiving cadaveric kidney transplantation [14,15,16,17]. In Japan, haemodialysis is the most common choice for ESRD treatment, followed by peritoneal dialysis and kidney transplantation [18]. Very few cadaveric kidney transplants are performed, and almost 90% of recipients receive living-donor kidney transplants from family members [19]. However, PEKT still accounts for 10% of kidney transplantations in Japan compared to 27% for the United States [18].

One of the most important tasks for patients with ESRD is to select their treatment modality. It was previously recommended that the process of selecting a kidney replacement therapy—haemodialysis, peritoneal dialysis, or kidney transplantation—should be shared between the patients, their family members, and medical professionals in a multidisciplinary kidney team [20,21]. According to clinical evidence, a multidisciplinary kidney team can provide detailed information on kidney replacement therapies, prior to progression to ESRD or the induction of dialysis [7]. Despite providing educational consultation programmes for shared decision-making in kidney replacement therapy, prior to starting kidney replacement therapy, most patients feel un-informed and that they do not have another option [22,23]. Several clinical studies highlighted discrepancies between a rational understanding and emotional lack of acceptance in decision-making among recipients of PEKT. PEKT is physically more advantageous than PDKT, but the mental satisfaction of recipients who received PEKT is less than that of those who received PDKT [24,25]. Surprisingly, a portion of PEKT recipients who did not receive other kidney replacement therapies before receiving their kidney transplantation perceived the transplantation as an event that deteriorated their well-being [26]. Based on these previous findings, patients with ESRD rationally understood the medical information provided by the medical professionals in the multidisciplinary kidney team, but could not emotionally accept that information. Therefore, the medical professionals in the multidisciplinary kidney team should recognize that clinical factors present obstacles to shared decision-making in kidney replacement therapy. Whereas, the incongruent perceptions of patients with ESRD suggest the possibility that such barriers might also be dependent upon factors related to the patients [27]. The discrepancy between rational a understanding and emotional lack of acceptance among recipients of kidney transplants suggests that a lack of independence might be involved in the decision-making processes of the recipients.

Indeed, chronic kidney disease has a high risk of comorbidity with several neuropsychiatric disorders, such as depression and anxiety disorders, since patients with chronic kidney disease suffer from distress due to continuous various restrictions and long-term medication [28]. Emotional disturbances, such as anxiety or depressive mood/perception can possibly impair and prolong the shared decision-making process, leading to blame being placed on the healthcare team [28,29]. It was previously established that the most important factors for patients when choosing a kidney replacement therapy is their own ability to express their personal values and beliefs based on independence and flexibility [22,30]. During the shared decision-making process for living-donor kidney transplantation, the medical staff in the multidisciplinary kidney team will already possess the ability to detect the sociopsychological abnormalities of patients with ESRD. Therefore, we speculate that there may be significant deficiencies in the current understanding of kidney transplantation strategies that provide the best kidney replacement therapy for patients with ESRD. When patients with ESRD select dialyses as their kidney replacement therapy, their family members are forced to change their lifestyles due to temporal and physical restraints. Conversely, when patients with ESRD select family-to-family living-donor kidney transplantation, a family member must donate his or her own kidney. These negative-to-negative psychological conflicts among patients with ESRD and their family members, when choosing between dialysis and transplantation can drastically affect familial psychodynamics. However, the detailed roles of candidate-donor family members in choosing family-to-family living-donor kidney replacement therapy remains to be clarified. Based on the above clinical questions, the present study determined the interactions between the sociopsychological factors (QOL and mental condition) of donors and recipients when choosing between PEKT and PDKT, to explore the fundamental impact factors on the decision-making process. 

## 2. Materials and Methods

### 2.1. Subjects

The subjects included 45 recipient/donor pairs (90 individuals) of PEKT (without receiving any kidney replacement therapies) and PDKT (receiving haemodialysis) at the Organ Transplantation Centre in Mie University Hospital from December 2016 to December 2019. The patients’ clinical data were obtained from medical records provided during the educational programme for living-donor kidney transplantation. All patients provided written informed consent before enrolment. The Institutional Review Board of Mie University School of Medicine approved this study protocol (Permission Number: 1606). Computation of a minimum required sample size was not statistically feasible for this study to observe the perceived QOL and psychological factors in choosing PEKT. Indeed, the choice of PEKT is complex and novel for this disease, and we had no preliminary data or any clear expectations for the results. Therefore, based on our lack of expectations, all pairs of donors and recipients who underwent kidney transplantation from 2016 to 2019 at our institute were considered target subjects.

### 2.2. Scale

To explore the factors affecting the selection of living-donor kidney transplantation, the QOL and mental health of the recipients and donors were determined using the Short Form Health Survey-36 version 2 (SF-36v2) [10,31,32], Profile of Mood States (POMS) [10,33,34,35] and State–Trait Anxiety Inventory (STAI) [36] at the formal choice of living-donor kidney transplantation, including PEKT or PDKT. 

SF-36v2 is a validated tool for measuring health-related QOL [10,31,32]. This tool consists of 8 subscales representing various health dimensions: “Physical functioning” (SF-36v2-PF), ”role physical” (SF-36v2-RP), “bodily pain” (SF-36v2-BP), “general health” (SF-36v2-GH), “vitality” (SF-36v2-VT), “social functioning” (SF-36v2-SF), “role emotional” (SF-36v2-RE) and “mental health” (SF-36v2-MH). These subscales can be used to generate summary component scores for physical (SF-36v2-PCS), mental (SF-36v2-MCS) and role/social (SF-36v2-RCS) QOL. Higher scores indicate a higher QOL. In this study, the raw data were converted into a standardized score for the healthy Japanese population by using a scoring algorithm (iHope International Inc., Kyoto, Japan). Clinically, a standardisation score > 40 is considered normal compared to the healthy general Japanese population [10,31].

The Profile of Mood States (POMS) includes 65 items rated on a five-point Likert scale. Participants can select a number from 0 (not at all) to 4 (extremely). Fifty-eight of the original items yielded scores for six factors: “tension/anxiety” (POMS-TA), “depression” (depressed mood: POMS-D), “anger/hostility” (POMS-AH), “vigour” (POMS-V), “fatigue” (POMS-F) and “confusion” (POMS-C) [10,33,34].

The State–Trait Anxiety Inventory (STAI) consists of 40 items for state anxiety (STAI-S: 20 items) and trait anxiety (STAI-T: 20 items). Items are answered on a 4-point Likert scale ranging from 1 (not at all) to 4 (very likely), each of which is divided into a range of 20 to 80, with higher scores indicating higher levels of anxiety symptoms [36].

### 2.3. Statistical Analysis

To explore the differences in QOL, mood and anxiety states between recipient and donor in PEKT vs. PDKT, scores of SF-36v2, POMS and STAI were analysed during kidney transplantation decision-making via analysis of variance (ANOVA) using BellCurve for Excel ver. 3.2 (Social Survey Research Information Co., Ltd., Tokyo, Japan). As the F-value of ANOVA was significant, the data were analysed using Scheffe’s post-hoc test. To explore the direct impact factors on decision-making between PEKT and PDKT, a binomial logistic regression analysis with robust standard errors was adopted using the free statistical software HAD version 17 (Shimizu, H., Kansei Gakuin University, Nishinomiya, Hyogo) (https://osf.io/32cyp/files/, accessed on 1 March 2021) [37,38]. To explore the secondary impact factors on the detected direct impact factors for decision-making, a stepwise multiple regression analysis with robust standard errors (HAD17) and analysis of covariance (ANCOVA) (BellCurve for Excel) were adopted. Multicollinearity was suspected in the binomial logistic regression and stepwise multiple regression analyses with robust standard errors, if the variance inflation factor (VIF) value was greater than 10 [37,39,40].

The background data of donors and recipients in PEKT and PDKT were analysed by a two-way ANOVA (age), a Mann–Whitney U test (duration of chronic kidney disease), a Cochran–Armitage test (relationship and primary disease), and the Cochran–Mantel–Haenstzel method (gender and employment) using BellCurve for Excel.

## 3. Results

### 3.1. Backgrounds

The subjects of this study included 24, and 21 pairs of PEKT and PDKT recipients (a total of 45 pairs), respectively. According to the ethical guidelines of the Japan Society for Transplantation [41], the Organ Transplantation Centre in Mie University Hospital limits living-donor kidney transplant donors to spouses and third-degree relatives. The detailed backgrounds of the subjects are summarized in Table 1. There were no differences of duration in chronic kidney disease between the PEKT and PDKT recipients (15.7 ± 15.3 and 11.4 ± 9.93 years, respectively). There were also no significant differences in other background factors between PEKT and PDKT (Table 1). 

### 3.2. QOL between Recipients and Donors in PEKT and PDKT

**Figure 1:**
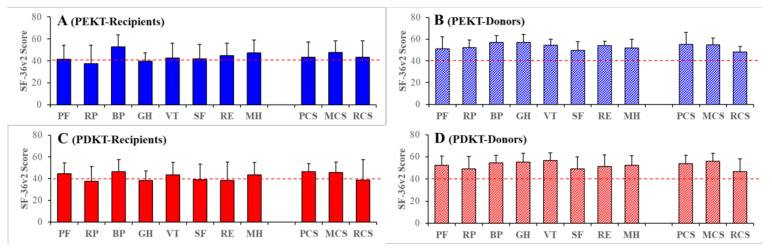
Short Form-36 Health Survey version 2 (SF-36v2) scores (quality of life: QOL) of recipients (**A**,**C**) and donors (**B**,**D**) in preemptive kidney transplantation (PEKT: **A**,**B**) and post-dialysis kidney transplantation (PDKT: **C**,**D**). Ordinates indicate the mean ± SD of the scores of SF-36v2. Red lines indicate the lowest SF-36v2 scores among the healthy general Japanese population. SF-36v2 is composed of eight subscales—“physical functioning” (PF), “role physical” (RP), “bodily pain” (BP), “general health” (GH), “vitality” (VT), “social functioning” (SF), “role emotional” (RE) and “mental health” (MH)—and three QOL components: Physical (PCS), mental (MCS), and role social (RCS) components.

Several previous studies reported that the QOL and mental conditions of patients and their caregivers/families were affected negatively by chronic kidney disease and haemodialysis [42,43,44]. Clinically, scores lower than 40 for SF-36v2 are considered to indicate deterioration of QOL compared to the Japanese general population [10,31]. Therefore, SF-36v2 scores of role physical (SF-36v2-RP) of PEKT (37.5 ± 16.9), PDKT (37.4 ± 13.7), and general health (SF-36v2-GH) of PEKT (39.6 ± 7.7) and PDKT (38.4 ± 8.7) of recipients were slightly different (low score) from those of the healthy general Japanese population [31]. In PDKT recipients, the SF-36v2 scores of role emotion (SF-36v2-RE: 38.4 ± 16.6) and social functioning (SF-36v2-SF: 38.9 ± 14.7) were also slightly different (low score) compared to the healthy general Japanese population [31]. However, no abnormalities in any other SF-36v2 factors were detected [31] (Figure 1). 

**Figure 2:**
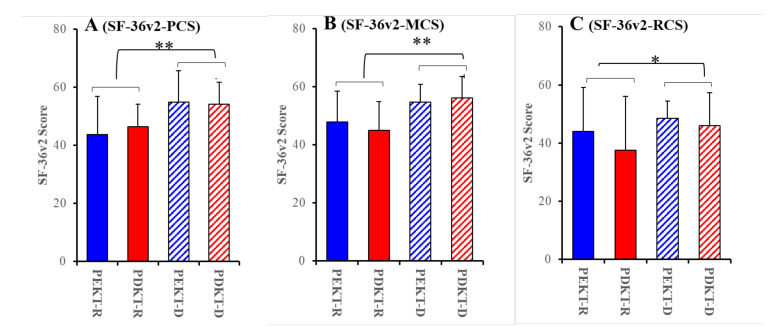
Comparisons of SF-36v2-PCS (**A**), SF-36v2-MCS (**B**), and SF-36v2-RSC (**C**) among PEKT recipients (PEKT-R), PDKT recipients (PDKT-R), PEKT donors (PEKT-D), and PDKT donors (PDKT-D) during kidney transplant decision-making. Ordinates indicate the mean ± SD for the scores of the SF-36v2 components. * *p* < 0.05, ** *p* < 0.01, relative to the SF-36v2 component scores of donors using a two-way analysis of variance (ANOVA) with Scheffe’s post-hoc test. Analyses between PEKT and PDKT were impossible since the F-values of the two-way ANOVA for the transplantation factor (PEKT vs. PDKT) and interaction factors (transplantation with relationship) were not violated (*p* > 0.05).

The two-way ANOVA detected a significant reduction in the recipients’ scores for all three SF-36v2 components, as well as SF-36v2-PCS [F_transplantation_(1, 89) = 0.02 (*p* > 0.1), F_relationship_(1, 89) = 20.39 (*p* < 0.01), F_transplantation*relationship_(1, 89) = 1.41 (>0.1)], SF-36v2-MCS [F_transplantation_(1, 89) = 0.01 (*p* > 0.1), F_relationship_(1, 89) = 20.58 (*p* < 0.01), F_transplantation*relationship_(1, 89) = 1.24 (>0.1)] and SF-36v2-RCS [F_transplantation_(1, 89) = 1.00 (*p* > 0.1), F_relationship_(1, 89) = 4.74 (*p* < 0.05), and F_transplantation*relationship_(1, 81) = 0.42 (>0.1)], compared to those of the donors (Figure 1 and Figure 2). Similar to the component scores, all 8 subscale scores of the recipients were lower than those of the donors (Figure 1). Notably, we did not detect any significant differences in QOL between the PEKT and PDKT pairs (Figure 1 and Figure 2). 

These results suggest that the QOL of recipients deteriorated compared to that of the donors. Contrary to our expectations, the QOL scores between the PEKT and PDKT pairs were almost equal. Notably, at the Organ Transplantation Centre of Mie University Hospital, the QOL of PEKT and PDKT recipients associated with role physical and general health slightly deteriorated. Whereas, the overall QOL of recipients generally remained almost equal to that of the general Japanese population. 

### 3.3. Mental State between Recipients and Donors of PEKT and PDKT

**Figure 3:**
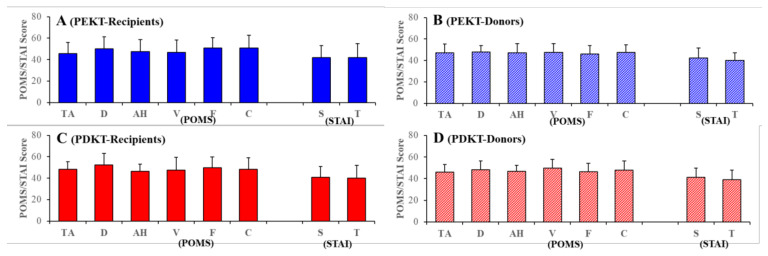
Scores for the Profile of Mood States (POMS) and State–Trait Anxiety Inventory (STAI) for the recipients (**A**,**C**) and donors (**B**,**D**) of PEKT (**A**,**B**) and PDKT (**C**,**D**). Ordinates indicate the mean ± SD of the scores of POMS and STAI. POMS is composed of 6 subscales: “tension/anxiety” (TA), “depression” (D), “anger/hostility” (AH), “vigour” (V), “fatigue” (F) and “confusion” (C). STAI is composed of two subscales: “state anxiety” (S) and “trait anxiety” (T).

The present study did not detect any psychopathological disturbances (POMS and STAI scores) in the PEKT and PDKT pairs or the correlations of mental state between donor–recipient and PEKT–PDKT using a two-way ANOVA (Figure 3). Furthermore, using a one-way ANOVA, we detected no differences in the scores of POMS and STAI for donors and recipients in PEKT and PDKT compared to the healthy general Japanese population [45]. Based on the results of SF-36v2, POMS, and STAI, the living-donor kidney transplantation pairs did not show psychosocial disturbances. Thus, no differences in psychosocial states were observed between the PEKT and PDKT pairs.

### 3.4. Impacts of the QOL and Mental States of Donors and Recipients on Decision-Making between PEKT and PDKT (Model 1)

In the above analyses (in Section 3.2 and Section 3.3), ANOVA did not detect any definitive QOL or mental conditions associated with choosing between PEKT and PDKT. Therefore, we subsequently explored the impact factors of QOL (SF-36v2) and mental state (POMS and STAI) on choosing between PEKT and PDKT using binomial logistic regression analysis with robust standard errors. 

All components of the SF-36v2 for donors and recipients did not affect decision-making result for PEKT and PDKT (Table 2). Contrary to the SF-36v2 components, binomial logistic regression analysis detected several significant impact factors. For recipients, decreasing the SF-36v2-RP score shifted the decision-making to PEKT, whereas decreasing the SF-36v2-SF and SF-36v2-RE scores shifted the decision-making to PDKT (Table 3). For donors, increasing the SF-36v2-VT score shifted the decision-making to PDKT, while increasing the SF-36v2-RP and SF-36v2-MH scores shifted the decision-making to PEKT (Table 3). 

Binomial logistic regression analysis detected a significant impact of the POMS factor among recipients when choosing between PEKT and PDKT, but did not detect any POMS factors for donors. Increasing the POMS-TA and POMS-D scores of the recipients shifted the decision-making to PDKT, while increasing the POMS-F and POMS-C of recipients shifted the decision-making to PEKT (Table 4). Unlike POMS, neither the STAI-S nor the STAI-T of both the donors and recipients affected their decision-making (Table 5). Therefore, the mental states of the donors did not affect decision-making directly. However, tension/anxiety and depressive mood among recipients contributed to choosing PDKT, while fatigue and confusion contributed to choosing PEKT.

### 3.5. Impacts of Mental States of Donors and Recipients on the Directly Impact Factors on Choosing between PEKT and PDKT (Model 2)

In the above analysis (in Section 3.4), we detected the direct impact factors on choosing between PEKT and PDKT using binomial logistic regression analysis. We explored the secondary/indirect impact factors on the direct impact factors of PEKT and PDKT using multiple regression analysis with robust standard errors. 

Increasing the POMS-TA of recipients directly shifted the decision-making to PDKT (Table 4) and additionally shifted the decision-making to PDKT via a reduction of the SF36v2-SF score (Table 6). Conversely, increasing POMS-F directly shifted the decision-making to PEKT (Table 4) but additionally shifted the decision-making to PDKT via a reduction of the SF36v2-RE score (Table 6). POMS-AH did not directly affect decision-making between PEKT or PDKT, but increasing the POMS-AH scores of recipients shifted the decision-making to PDKT by increasing SF36v2-RP and to PEKT by additionally increasing both the SF36v2-SF and SF36v2-RE scores (Table 6). Similar to the recipients, POMS-AH did not directly affect decision-making but increasing the POMS-AH of donors additionally shifted their decision-making to PEKT by increasing the SF36v2-MH scores (Table 6). 

Contrary to POMS, increasing the STAI-T of recipients increased the scores of POMS-TA, POMS-D, POMS-AH, and POMS-C (Table 7). The STAI-T of the donors selectively increased the POMS-AH score (Table 7). The STAI-S scores of neither the donors nor the recipients affected any POMS scores based on the multiple regression analysis (data not shown). 

### 3.6. Impacts of the State Anxiety of Donors and Recipients on Mental Condition (Model 3)

Based on the results of the multiple regression analysis, STAI-T affected various direct/secondary impact POMS factors. To explore the contradictive effects of STAI-T (trait anxiety) on decision-making, the relationship between POMS scores (POMS-TA, POMS-D, POMS-AH, and POMS-C) and STAI-T was analysed using ANCOVA. 

**Figure 4:**
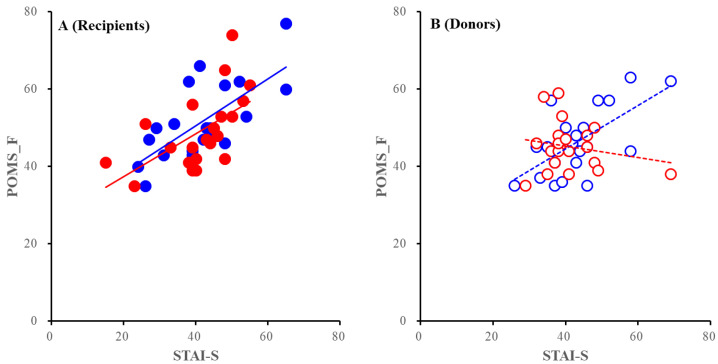
Correlation between the POMS-TA and STAI-T of recipients (**A**) and donors (**B**). Blue and red circles indicate PEKT, and PDKT, respectively. Closed and opened circles indicate recipients and donors, respectively. Full and dotted lines indicate the regressions of recipients and donors, respectively. Ordinates and abscissas indicate the mean ± SD of the POMS-TA, and STAI-T scores, respectively.

No significant differences were detected in the correlation between POMS-C, POMS-D, POMS-AH, and STAI-T in any combinations (data not shown). In relation to the correlation between POMS-TA and STAI-T, ANCOVA did not detect significant differences among recipients (PEKT vs. PDKT) or PDKT (donors vs. recipients), but the POMS-TA scores of the PEKT donors were less sensitive to STAI-T than those of the PDKT donors [F_transplantation: fixed factor_(1, 44) = 3.906 (*p* > 0.05), F_STAI-T: covariance factor_ (1, 44) = 7.237 (*p* < 0.05), F_transplantation*STAI-T_ (1, 44) = 4.697 (*p* < 0.05)] (Figure 4). The reactive tension/anxiety (POMS-TA) among PEKT donors likely indicates greater tolerability to trait anxiety in this group compared to that of the PDKT donors (Figure 4).

## 4. Discussion

### 4.1. Interactions between QOL and Mental States of Recipients and Donors in Choosing between PEKT or PDKT

**Figure 5:**
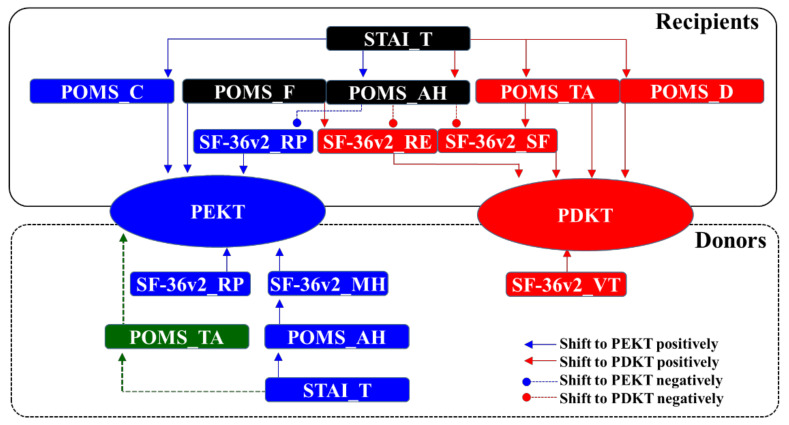
Proposed cascades of decision-making processes for PEKT and PDKT.

The present study revealed the impacts of various psychosocial features and conditions of recipients and donors on decision-making between PEKT and PDKT. However, the decision-making processes were composed of more complicated interactions between psychosocial factors than we expected. Figure 5 presents the psychosocial interactions revealed among recipients and donors in the decision-making processes of PEKT and PDKT. 

Binomial logistic regression with robust standard errors detected the various direct impact factors of recipients, showing that low recipient scores of SF-36v2-RP (role physical) contributed to choosing PEKT, whereas low recipient scores of SF-36v2-RE (role emotional) and SF-36v2-SF (social functioning) shifted the decision-making to PDKT (Table 3 and Figure 5). The mental states of the recipients also directly contributed to their decision-making processes. High scores in POMS-C (confusion) and POMS-F (fatigue) among recipients directly shifted the decision-making to PEKT. Whereas, high scores of POMS-TA (tension/anxiety) and POMS-D (depression) among recipients directly shifted the decision-making to PDKT (Table 4 and Figure 5). Multiple regression with robust standard errors also detected secondary factors (Table 6 and Table 7 and Figure 5), indicating that a high score in POMS-TA additionally shifted the decision-making to PDKT via a reduction of the SF-36v2-SF scores. However, high POMS-F scores directly shifted the decision-making to PEKT but biphasically shifted the decision-making to PDKT via a reduction of the SF-36v2-RE score. Interestingly, POMS-AH (anger/hostility) provided secondary negative factors for decision-making of PEKT and PDKT by increasing the SF-36v2-RP, SF-36v2-RE, and SF-36v2-SF scores. The present study did not detect any direct impacts of STAI scores on decision-making, whereas STAI-T (trait anxiety) indirectly affected the decision-making processes of both PEKT and PDKT. 

Unlike the recipients, the donors’ mental states and POMS and STAI scores did not directly affect decision-making. Binomial logistic regression analysis also detected direct impact factors among donors, showing that high donor scores SF-36v2-VT (vitality) contributed to choosing PDKT. Whereas, high donor scores of SF-36v2-RP (role physical) and SF-36v2-MH (mental health) shifted the decision-making to PEKT. High scores of POMS-AH (anger/hostility) and STAI-T (trait anxiety) of donors indirectly shifted the decision-making to PEKT via a increasing SF-36v2-MH scores (Table 6 and Table 7 and Figure 5). 

### 4.2. Importance of Life–Work–Family Balance for Patients with ESRD and Their Families When Deciding on a Kidney Replacement Therapy

The results of this study suggest that the donors rationally decided to donate their kidneys to the recipients (patients with ESRD) to maintain the QOL of their family members. The recipients also rationally decided to accept the kidney transplants, but the effects of psychological factors on decision-making cannot be ignored [46]. Indeed, patients who will soon reach ESRD demand a large amount of information, not only from the multidisciplinary kidney team, but also for other patients with experience regarding dialyses and transplantation. This is because patients must discuss their choices with their family members and colleagues, prior to the initiation of kidney replacement therapy, due to subsequent changes in patients’ lifestyles [30]. This presents serious life–work–family balance issues for patients and their family members [37,39,40,47,48,49]. According to the definition of Grzywacz and Carlson [47], work–family balance is the “accomplishment of role-related expectations that are negotiated and shared between individuals and their role-related partners in the work and family domains”. Work–family enrichment is considered “the extent to which experience in one role improves the QOL in the other role” [48]. Work–family conflict is considered “a form of inter-role conflict in which the demands of work and family roles are incompatible in some respect so that participation, in either the work or family role is more difficult because of participation in the other role” [49].

Taken together with the life–work–family balance concept, the present results indicate the rational clinical value of decision-making for kidney replacement therapy, but also the importance of maintaining one’s lifestyle and the QOL of patients with ESRD, as well as that of their family members. According to the decision-making process (Figure 5), donors decided to donate their kidneys to maintain their lifestyles and QOL, but recipients seemed to accept kidney transplantation to compensate for their loss of function. These interactions between donors and recipients in the decision-making process for family-to-family living-donor kidney transplantation suggest that the proactive persuasion of donors plays an unexpectedly important role in decision-making for transplantation. Indeed, the mental states of recipients with PEKT and PDKT at kidney transplantation decision-making were predominantly confusion, and tension/anxiety/depression, respectively.

The proactive donor stance and passive recipient stance seem to contradict common clinical observations. Generally, in Japan, to protect candidate donors, it is obligatory for third parties to evaluate the validity and reliability of donor’s decisions when deciding to donate a kidney [41]. According to the ethical guidelines of the Japan Society for Transplantation [41], the Organ Transplantation Centre in Mie University Hospital also evaluates the validity/reliability of the donor’s decision. Transplantation coordinators, transplantation surgeons, psychologists and psychiatrists must evaluate the validity/reliability of the decision-making of donors and recipients at multiple stages during the shared decision-making processes. The multi-stage evaluations of the validity/reliability of decision-making processes for both donors and recipients confirmed that neither would force a kidney transplant on the other party. Therefore, the positive impacts of POMS scores for decision-making among PEKT (POMS-C) and PDKT (POMS-TA and POMS-D) recipients, as detected by binomial logistic regression analysis, likely detected the recipients’ psychological reactions to decision-making, rather than the impact factors for decision-making. 

### 4.3. Impact of Mental State on Choosing between PEKT and PDKT

It is well-known that anxiety plays important roles in the psychological and biological risk factors for the impairment of emotional perception/cognition [50]. The trait anxiety of the recipients enhanced negative mental responses (POMS-TA/POMS-D and POMS-C) in the selection between PDKT, and PEKT, respectively. Confusion and anxiety/tension/depression are emotional/cognitive impairments that are unfavourable for independent rational decision-making. A recent clinical study reported a high prevalence of irritability (46.7%); abnormal illness behaviour, including anxiety (23.3%); and somatization (23.3%) among patients waitlisted for cadaveric kidney transplantation using the Diagnostic Criteria for Psychosomatic Research (DCPR) [50]. Notably, among patients with a negative score based on the International Classification of Diseases version 10, 77.8% patients exhibited DCPR syndrome [50]. These findings are consistent with those of other studies, which frequently reported on the association between psychological disturbances and various other physical diseases among patients [10,34,51]. Therefore, the tendencies shown in this study are understandable, as patients with ESRD are often worried about the unknown factors of their condition, based on a fear that they may lose their renal functions and experience other unexpected outcomes [10,34,50,51].

The present study demonstrated the additional importance of the impacts from individually acquired trait anxiety, beyond the traditional clinical concept. The chronic progress features of chronic kidney disease were shown to play important roles in the psychopathology of depression and anxiety observed in patients with ESRD. STAI can measure state anxiety (anxiety that occurs transiently under specific conditions) and trait anxiety (long-term anxiety unaffected by contextual factors) [36]. Traditionally, trait anxiety has been regarded as a construct that signifies the underlying causes of the various thoughts, feelings, and behaviours that correspondingly reflect the presence of anxiety. However, the recent network theory of personality defines trait anxiety as a formative construct emerging from interactions among its constitutive features (e.g., thoughts, feelings, and behaviours), but not a latent cause of these features [52]. The present study did not detect the direct impact of STAI-T (trait anxiety) on decision-making since the STAI-T score significantly increased the factors of decision-making for both PEKT and PDKT, such as POMS-C, POMS-AH, POMS-TA, and POMS-D. The various contradictive impacts of the recipient’s trait anxiety on decision-making processes suggest that recipients vulnerable to anxiety cannot independently make a decision on their kidney replacement therapy. Recipients with a tendency to experience increased anxiety/tension and depressive mood indue to distress avoid kidney transplantation decision-making as a psychological defence mechanism, resulting in choosing dialyses. Whereas, recipients with a tendency to experience increased confusion due to distress shift their decision-making to PEKT under the proactive persuasion of their family members (candidate donors). Therefore, STAI-T does not provide direct decision-making factors but instead enhances the direct or secondary factors of decision-making.

Unlike those of the recipients, the donors’ mental state, POMS, and STAI scores did not directly affect the decision-making processes for either PEKT or PDKT. Binominal logistic regression analysis detected direct impact factors of donors, where high donor SF-36v2-VT scores (vitality) contributed to choosing PDKT, while high donor scores for SF-36v2-RP (role physical) and SF-36v2-MH (mental health) shifted the decision-making to PEKT. These results suggest that donors rationally decide to donate their kidneys based to maintain their lifestyles and the QOL of their family members without affecting their emotional states. Furthermore, a high POMS-AH score (anger/hostility) shifted the choice to PEKT by increasing the SF-36v2-MH score. The donors’ reactions in the decision-making processes for PEKT possibly represent a response to disadvantaging their family members (patients) and themselves. Furthermore, the ANCOVA analysis detected an interesting relationship between tension/anxiety and trait anxiety in the decision-making processes of PEKT donors. PEKT donors were less sensitive to their trait anxiety (STAI-T) than the donors of PDKT (Figure 4). Taken together with the tendencies of PEKT recipients, the supportive/proactive interventions of donors to aid confused patients with ESRD seem to play unexpectedly fundamental roles in the decision-making processes for PEKT. The present study detected only one direct impact factor among PDKT donors: High scores of donor SF-36v2-VT (vitality) contributed to choosing PDKT without any secondary mental conditioning factors, indicating that the PDKT donors chose PDKT due to a mature and rational decision-making process. Although we did not add the date at which the PDKT donors decided to donate their kidneys as a factor in this quantitative study, at the final interview to evaluate the validity/reliability of donor decisions, the majority of PDKT donors clearly stated that they had waited until the recipient decided to accept the donor’s kidney. This result indicates that PDKT donors expressed their intention to donate their kidneys but could not supportively/proactively persuade patients with ESRD to make rational decisions regarding their kidney replacement therapy. In other words, donors withholding proactive confirmation of their decisions (to donate their kidneys) likely leads to the induction of dialyses among patients with ESRD due to their hesitance to select family-to-family living-donor kidney transplantation. Therefore, compared to PEKT donors, PDKT donors, who exhibit vulnerability to enhanced tension/anxiety induced by trait anxiety, require more vitality for proactive persuade to accept transplantation from patients with ESRD. Additionally, changing the current kidney replacement therapy being received to another modality entails a mental burden and requires vitality.

The most recent focus in kidney transplant medicine involves determining when to perform a transplant. The risk of delayed graft functions in PEKT range from 2 to 4%, whereas the risk in PDKT is 4–10% [53,54,55,56]. Previous clinical findings indicate that PEKT is a better option than PDKT in terms of physical prognosis. However, PEKT should not be recommended only because of its advantages in physical prognosis. Recent Japanese studies revealed that there is no difference in long-term QOL prognoses between PEKT and PDKT recipients. Surprisingly, recipients’ mental satisfaction with PDKT was found to be higher than that with PEKT [24,25]. PEKT is physically more advantageous than PDKT, but PDKT seems to be no worse than PEKT in its long-term prognosis of QOL and the development of health literacy and adherence. The discrepancies in satisfaction between PEKT and PDKT recipients seem to indicate the impacts of ameliorating the distress associated with dialyses. However, the influence of confusion among PEKT recipients demonstrated in this study must not be ignored. Patients with low health literacy generally have worse outcomes, as well as a lower chance of being truly involved in their decision-making [57]. Clearly, cognitive and perceptual vulnerabilities due to trait anxiety can negatively affect the health literacy of individuals. It is also well-known that patients with chronic physical diseases, including ESRD treated with dialysis are at a high risk of suicide and comorbidities with affective disorders [10,34,37,58], since haemodialysis, which requires time and physical restraints, forces patients and their families to change their lifestyles. Therefore, enriching the independence of PEKT recipients during the shared decision-making process would likely improve satisfaction, adherence, and the development of health literacy. Enriching a recipient’s independence and life–work–family balance would likely require long-term educational consultation efforts.

To further improve the QOL of patients with ESRD and their families, based on the present results, we propose the following areas of focus: When should one perform kidney transplantation, and when should one start and continue educational consultation programmes for shared decision-making in kidney replacement therapy according to the physical and sociopsychological prognoses? Professional staff in multidisciplinary kidney teams for shared decision-making programmes should employ clinical evidence along with our proposed focus areas based on the physical and sociopsychological prognoses. 

## 5. Conclusions

The present study determined the impact of the QOL, and the mental states of donors and recipients in choosing to undergo family-to-family living-donor kidney transplantation, including PEKT and PDKT, at the point in time when patients finalise their decisions in that respect. The purpose of the study was to further improve the psychosocial prognoses of donor/recipient pairs through educational consultation programmes. The analyses in this study indicated that the decision-making process of both PEKT and PDKT were unexpectedly reliant on the proactive/independent interventions of the donors, rather than the independent choices of the recipients. The present study detected only negative/passive factors among the recipients, including the impacts of tension/anxiety, depressive mood, fatigue, and confusion, which negatively affected the decision-making processes of kidney replacement therapy. We also detected the typical features of PEKT donors, including high tolerability against the trait anxiety of reactive tension/anxiety compared to that of the PDKT donors. Contrary to our expectations, for patients with ESRD, these results suggest that the proactive/supportive intervention of family members (candidate donors) likely plays a fundamental role in kidney transplantation decision-making (PEKT). Additionally, the maturation of life-work-family balance concepts of donors is required to change current kidney replacement therapy from dialyses to transplantation (PDKT). Therefore, to facilitate shared decision-making in kidney replacement therapy, medical professionals in the multidisciplinary kidney teams should provide detailed information to patients with ESRD and their families as potential donors through educational consultation programmes before the induction of kidney replacement therapy and during dialyses.

## Figures and Tables

**Table 1:** Background of subjects in this study. PEKT: Pre-emptive kidney transplantation. PDKT: post-dialysis kidney transplantation. The statistical *p* values were analysed using a two-way ANOVA (age), a Mann–Whitney U test (duration of chronic kidney disease), a Cochran–Armitage test (relationship and primary disease), and the Cochran–Mantel–Haenstzel method (gender and employment).

	PEKT		PDKT	
	Recipient	Donor	Recipient	Donor
Male/Female	13/11	8/16	14/7	9/12
Age	50.4 ± 12.1	59.9 ± 10.4	52.0 ± 13.5	60.1 ± 12.4
Employment/Unemployment	14/10	17/7	16/5	18/3
Duration of chronic kidney disease (years)	15.7 ± 15.3		11.4 ± 9.9	
Relationship				
spouse	16(67%)		9(43%)	
offspring	7(29%)		9(43%)	
sibling	1(4%)		1(5%)	
father in law	0		1(5%)	
brother in law	0		1(5%)	
Primary disease				
unknown chronic renal failure	8(33%)		1(5%)	
diabetic nephropathy	2(8%)		6(29%)	
polycystic kidney disease	5(21%)		1(5%)	
glomerulonephritis	3(13%)		1(5%)	
IgA nephropathy	1(4%)		3(14%)	
lupus nephritis	1(4%)		2(10%)	
Others	4(16%)		6(29%)	

**Table 2:** Impact components of SF-36v2 on choosing between PEKT and PDKT for recipients and donors analysed using binomial logistic regulation analysis with robust standard errors. β means standard partial regression coefficient. SE: standard Error. VIF: Variance Inflation Factor. OR: Odds Ration.

Nagelkerke R^2^ (*p* Value)	Components		β	SE	*p* Value	VIF	OR	OR (95% CI)	
0.078 (0.872)	Recipients	SF-36v2_PCS	0.025	0.029	0.933	1.117	1.025	0.968	1.085
		SF-36v2_MCS	−0.013	0.035	0.393	1.165	0.987	0.922	1.057
		SF-36v2_RCS	−0.003	0.020	0.715	1.225	0.997	0.959	1.037
	Donors	SF-36v2_PCS	−0.022	0.038	0.881	1.105	0.978	0.908	1.053
		SF-36v2_MCS	0.038	0.053	0.550	1.229	1.038	0.936	1.152
		SF-36v2_RCS	−0.019	0.034	0.479	1.072	0.981	0.917	1.049

**Table 3:** Significant impact factors of SF-36v2 on choosing between PEKT and PDKT. * *p* < 0.05, ** *p* < 0.01 based on binomial logistic regulation analysis with robust standard errors. Multicollinearity was suspected if the VIF value was greater than 10. * *p* < 0.05, ** *p* < 0.01: significant impact factor on choosing PEKT or PDKT for recipients and donors.

Nagelkerke R^2^ (*p* Value)	Factors		β	SE	*p* Value	VIF	OR	OR (95% CI)	
0.763 (0.005 **)	Recipients	SF3-6v2_PF	0.145	0.081	0.072	2.044	1.157	0.987	1.355
		SF-36v2_RP	0.332	0.148	0.025 *	3.638	1.394	1.043	1.863
		SF-36v2_BP	−0.252	0.084	0.003 **	2.419	0.777	0.659	0.916
		SF-36v2_GH	0.115	0.069	0.096	2.277	1.122	0.980	1.284
		SF-36v2_VT	−0.023	0.099	0.815	3.519	0.977	0.805	1.186
		SF-36v2_SF	−0.094	0.055	0.090	3.859	0.911	0.817	1.015
		SF-36v2_RE	−0.178	0.100	0.076	3.163	0.837	0.688	1.019
		SF-36v2_MH	−0.069	0.116	0.555	3.625	0.934	0.743	1.173
	Donors	SF-36v2_PF	−0.044	0.078	0.577	2.273	0.957	0.821	1.116
		SF-36v2_RP	0.088	0.093	0.344	2.277	1.092	0.910	1.312
		SF-36v2_BP	−0.210	0.100	0.035 *	1.856	0.811	0.667	0.986
		SF3-6v2_GH	0.006	0.087	0.942	1.638	1.006	0.848	1.194
		SF-36v2_VT	0.373	0.168	0.026 *	2.706	1.452	1.045	2.018
		SF-36v2_SF	0.033	0.125	0.790	2.422	1.034	0.809	1.322
		SF-36v2_RE	−0.340	0.222	0.125	2.225	0.712	0.461	1.099
		SF-36v2_MH	0.003	0.126	0.979	4.943	1.003	0.784	1.283

The significant values of SF-36v2_SF and SF-36v2_RE in recipients and SF-36v2_MH in donors in the published version became non-significant values after binomial logistic regression analysis using corrected data.

**Table 4:** Significant impact factors of POMS on choosing between PEKT and PDKT. * *p* < 0.05 according to binomial logistic regulation analysis with robust standard errors. Multicollinearity was suspected if the VIF value was greater than 10. * *p* < 0.05: significant impact factor on choosing between PEKT and PDKT for recipients and donors.

Nagelkerke R^2^ (*p* Value)	Factor		β	SE	*p* Value	VIF	OR	OR (95% CI)	
0.679 (0.005 *)	Recipients	POMS_TA	0.396	0.194	0.041 *	5.667	1.485	1.015	2.173
		POMS_D	0.573	0.236	0.015 *	7.651	1.774	1.117	2.817
		POMS_AH	−0.194	0.132	0.141	5.063	0.824	0.636	1.066
		POMS_V	−0.036	0.080	0.655	2.167	0.965	0.824	1.129
		POMS_F	−0.512	0.238	0.032 *	3.978	0.600	0.376	0.956
		POMS_C	−0.336	0.141	0.017 *	5.500	0.714	0.542	0.942
	Donors	POMS_TA	−0.397	0.210	0.059	4.380	0.672	0.445	1.015
		POMS_D	0.013	0.161	0.936	3.305	1.013	0.739	1.388
		POMS_AH	−0.027	0.127	0.834	2.618	0.974	0.759	1.249
		POMS_V	0.078	0.089	0.382	1.487	1.081	0.907	1.288
		POMS_F	−0.036	0.130	0.785	1.800	0.965	0.748	1.246
		POMS_C	0.508	0.243	0.037 *	3.115	1.662	1.032	2.678

The non-significant value of POMS_C in donors in the published version became a significant value after binomial logistic regression analysis using corrected data.

**Table 5:** Impact factor of STAI on choosing between PEKT and PDKT detected according to binomial logistic regulation analysis with robust standard errors. Multicollinearity was suspected if the VIF value was greater than 10. * *p* < 0.05, ** *p* < 0.01: significant impact factor on choosing between PEKT and PDKT for recipients and donors.

Nagelkerke R^2^ (*p* Value)	Factor		β	SE	*p* Value	VIF	OR	OR (95% CI)	
0.067 (0.713)	Recipients	STAI_S	−0.022	0.059	0.706	3.861	0.978	0.872	1.097
		STAI_T	−0.001	0.049	0.977	3.835	0.999	0.907	1.099
	Donors	STAI_S	−0.012	0.040	0.768	1.376	0.988	0.914	1.069
		STAI_T	−0.057	0.053	0.283	1.395	0.945	0.852	1.048

**Table 6:** Significant impact factors of POMS on choosing between PEKT and PDKT. Multicollinearity was suspected if the VIF value was greater than 10. * *p* < 0.05, ** *p* < 0.01: significant effects of POMS scores on the direct impact factors for choosing between PEKT and PDKT among recipients and donors according to stepwise multiple regression analysis with robust standard errors.

Model		Adjusted R^2^	*F* Value	*p* Value	Factor	β	*p* Value
Recipient	SF-36v2_RP	0.288	6.015	0.001 **	POMS_AH	0.653	0.011 *
Donor	SF-36v2_BP	0.092	2.485	0.043 *	POMS_V	−0.257	0.049 *
					POMS_C	−0.430	0.005 **

According to the correction of results in Table 3, the statistical results by multiple regression analyses of SF-36v2_SF and SF-36v2_RE in recipients and SF-36v2_MH in donors were eliminated. The significant values of POMS_V and POMS_C for SF-36v2_BP in donors could be detected by multiple regression analysis using corrected data.

**Table 7:** Significant impact factors of STAI on choosing between PEKT and PDKT. Multicollinearity was suspected if the VIF value was greater than 10. * *p* < 0.05, ** *p* < 0.01: significant effects of STAI scores on the direct/secondary impact factors for choosing between PEKT and PDKT among recipients and donors according to stepwise multiple regression analysis with robust standard errors.

Model		Adjusted R^2^	*F* Value	*p* Value	Factor	β	*p* Value
Recipient	POMS_TA	0.443	14.062	0.001 **	STAI_T	0.516	0.018 *
	POMS_D	0.546	14.849	0.001 **	STAI_T	0.852	0.001 **
	POMS_AH	0.397	17.744	0.001 **	STAI_T	0.633	0.001 **
	POMS_C	0.439	18.830	0.001 **	STAI_T	0.822	0.001 **
Donor	POMS_C	0.174	4.067	0.025 *	STAI_T	0.478	0.025 *

According to the correction of results in Table 4 and Table 6, the statistical result by multiple regression analyses of POMS_AH in donors was eliminated. The significant values of STAI_T for POMS_C in donors could be detected by multiple regression analysis using corrected data (red painted factors).

## Data Availability

The data presented in this study are available on request from the corresponding author. The data are not publicly available due to equipment dependent data.
