# Correction: Hasegawa et al. Impacts of Interaction of Mental Condition and Quality of Life between Donors and Recipients at Decision-Making of Preemptive and Post-Dialysis Living-Donor Kidney Transplantation. *J. Pers. Med.* 2021, *11*, 414

**DOI:** 10.3390/jpm12122036

**Published:** 2022-12-09

**Authors:** Toshiki Hasegawa, Kouhei Nishikawa, Yuko Tamura, Tomoka Oka, Aiko Urawa, Saori Watanabe, Shugo Mizuno, Motohiro Okada

**Affiliations:** 1 Department of Neuropsychiatry, Division of Neuroscience, Graduate School of Medicine, Tsu 514-8507, Japan; 2 Organ Transplantation Centre, Mie University Hospital, Tsu 514-8507, Japan; 3 Department of Nephro-Urologic Surgery and Andrology, Mie University Graduate School of Medicine, Tsu 514-8507, Japan; 4 Department of Psychology, Graduate School of Nursing, Mie University, Tsu 514-8507, Japan

## Error in Tables 3, 4, 6 and 7 and Figure 4

The authors wish to make the following corrections to this paper [1]. The authors identified inadvertent errors in linking between anonymizing donors and recipients. Re-analyses of the corrected linking data indicated several different results compared to the published version. The corrected results are indicated in the following Table 3, Table 4, Table 6 and Table 7 and Figure 4. The authors declare that these improvements reflect a part of the overall results, and do not affect the importance of life–work–family balance in the decision-making process of transplantation. The authors would like to apologize for any inconvenience caused to the readers by these improvements.

**Table 3:** Significant impact factors of SF-36v2 on choosing between PEKT and PDKT. * *p* < 0.05, ** *p* < 0.01 based on binomial logistic regulation analysis with robust standard errors. Multicollinearity was suspected if the VIF value was greater than 10. * *p* < 0.05, ** *p* < 0.01: significant impact factor on choosing PEKT or PDKT for recipients and donors.

Nagelkerke R^2^ (*p* Value)	Factors		β	SE	*p* Value	VIF	OR	OR (95% CI)	
0.763 (0.005 **)	Recipients	SF3-6v2_PF	0.145	0.081	0.072	2.044	1.157	0.987	1.355
		SF-36v2_RP	0.332	0.148	0.025 *	3.638	1.394	1.043	1.863
		SF-36v2_BP	−0.252	0.084	0.003 **	2.419	0.777	0.659	0.916
		SF-36v2_GH	0.115	0.069	0.096	2.277	1.122	0.980	1.284
		SF-36v2_VT	−0.023	0.099	0.815	3.519	0.977	0.805	1.186
		SF-36v2_SF	−0.094	0.055	0.090	3.859	0.911	0.817	1.015
		SF-36v2_RE	−0.178	0.100	0.076	3.163	0.837	0.688	1.019
		SF-36v2_MH	−0.069	0.116	0.555	3.625	0.934	0.743	1.173
	Donors	SF-36v2_PF	−0.044	0.078	0.577	2.273	0.957	0.821	1.116
		SF-36v2_RP	0.088	0.093	0.344	2.277	1.092	0.910	1.312
		SF-36v2_BP	−0.210	0.100	0.035 *	1.856	0.811	0.667	0.986
		SF3-6v2_GH	0.006	0.087	0.942	1.638	1.006	0.848	1.194
		SF-36v2_VT	0.373	0.168	0.026 *	2.706	1.452	1.045	2.018
		SF-36v2_SF	0.033	0.125	0.790	2.422	1.034	0.809	1.322
		SF-36v2_RE	−0.340	0.222	0.125	2.225	0.712	0.461	1.099
		SF-36v2_MH	0.003	0.126	0.979	4.943	1.003	0.784	1.283

The significant values of SF-36v2_SF and SF-36v2_RE in recipients and SF-36v2_MH in donors in the published version became non-significant values after binomial logistic regression analysis using corrected data.

**Table 4:** Significant impact factors of POMS on choosing between PEKT and PDKT. * *p* < 0.05 according to binomial logistic regulation analysis with robust standard errors. Multicollinearity was suspected if the VIF value was greater than 10. * *p* < 0.05: significant impact factor on choosing between PEKT and PDKT for recipients and donors.

Nagelkerke R^2^ (*p* Value)	Factor		β	SE	*p* Value	VIF	OR	OR (95% CI)	
0.679 (0.005 *)	Recipients	POMS_TA	0.396	0.194	0.041 *	5.667	1.485	1.015	2.173
		POMS_D	0.573	0.236	0.015 *	7.651	1.774	1.117	2.817
		POMS_AH	−0.194	0.132	0.141	5.063	0.824	0.636	1.066
		POMS_V	−0.036	0.080	0.655	2.167	0.965	0.824	1.129
		POMS_F	−0.512	0.238	0.032 *	3.978	0.600	0.376	0.956
		POMS_C	−0.336	0.141	0.017 *	5.500	0.714	0.542	0.942
	Donors	POMS_TA	−0.397	0.210	0.059	4.380	0.672	0.445	1.015
		POMS_D	0.013	0.161	0.936	3.305	1.013	0.739	1.388
		POMS_AH	−0.027	0.127	0.834	2.618	0.974	0.759	1.249
		POMS_V	0.078	0.089	0.382	1.487	1.081	0.907	1.288
		POMS_F	−0.036	0.130	0.785	1.800	0.965	0.748	1.246
		POMS_C	0.508	0.243	0.037 *	3.115	1.662	1.032	2.678

The non-significant value of POMS_C in donors in the published version became a significant value after binomial logistic regression analysis using corrected data.

**Table 6:** Significant impact factors of POMS on choosing between PEKT and PDKT. Multicollinearity was suspected if the VIF value was greater than 10. * *p* < 0.05, ** *p* < 0.01: significant effects of POMS scores on the direct impact factors for choosing between PEKT and PDKT among recipients and donors according to stepwise multiple regression analysis with robust standard errors.

Model		Adjusted R^2^	*F* value	*p* Value	Factor	β	*p* Value
Recipient	SF-36v2_RP	0.288	6.015	0.001 **	POMS_AH	0.653	0.011 *
Donor	SF-36v2_BP	0.092	2.485	0.043 *	POMS_V	−0.257	0.049 *
					POMS_C	−0.430	0.005 **

According to the correction of results in Table 3, the statistical results by multiple regression analyses of SF-36v2_SF and SF-36v2_RE in recipients and SF-36v2_MH in donors were eliminated. The significant values of POMS_V and POMS_C for SF-36v2_BP in donors could be detected by multiple regression analysis using corrected data.

**Table 7:** Significant impact factors of STAI on choosing between PEKT and PDKT. Multicollinearity was suspected if the VIF value was greater than 10. * *p* < 0.05, ** *p* < 0.01: significant effects of STAI scores on the direct/secondary impact factors for choosing between PEKT and PDKT among recipients and donors according to stepwise multiple regression analysis with robust standard errors.

Model		Adjusted R^2^	*F* Value	*p* Value	Factor	β	*p* Value
Recipient	POMS_TA	0.443	14.062	0.001 **	STAI_T	0.516	0.018 *
	POMS_D	0.546	14.849	0.001 **	STAI_T	0.852	0.001 **
	POMS_AH	0.397	17.744	0.001 **	STAI_T	0.633	0.001 **
	POMS_C	0.439	18.830	0.001 **	STAI_T	0.822	0.001 **
Donor	POMS_C	0.174	4.067	0.025 *	STAI_T	0.478	0.025 *

According to the correction of results in Table 4 and Table 6, the statistical result by multiple regression analyses of POMS_AH in donors was eliminated. The significant values of STAI_T for POMS_C in donors could be detected by multiple regression analysis using corrected data (red painted factors).

**Figure 4:**
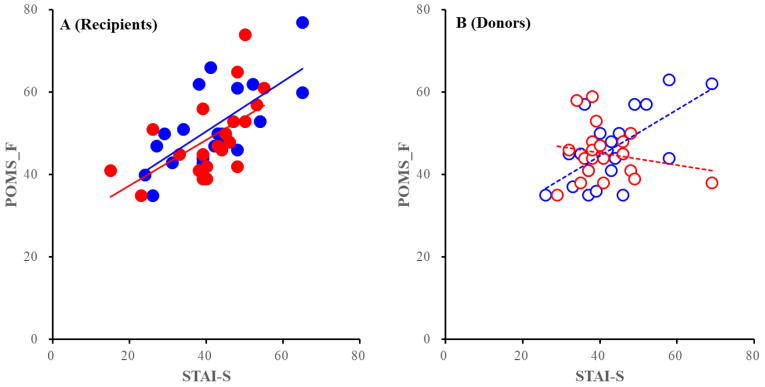
Correlation between the POMS-TA and STAI-T of recipients (**A**) and donors (**B**). Blue and red circles indicate PEKT, and PDKT, respectively. Closed and opened circles indicate recipients and donors, respectively. Full and dotted lines indicate the regressions of recipients and donors, respectively. Ordinates and abscissas indicate the mean ± SD of the POMS-TA, and STAI-T scores, respectively.

In the published version, POMS-TA in PEKT donors was less sensitive to STAI-T than in PDKT donors from the analysis of covariance (ANCOVA), but ANCOVA using corrected data could not detect this difference. On the contrary, POMS_F in PDKT donors was less sensitive to STAI-T than in PEKT donors by ANCOVA using corrected data.
